# Identification of RNA Markers in Red Blood Cells for Doping Control in Autologous Blood Transfusion

**DOI:** 10.3390/genes13071255

**Published:** 2022-07-15

**Authors:** Takehito Sugasawa, Yasuharu Kanki, Ritsuko Komine, Koichi Watanabe, Kazuhiro Takekoshi

**Affiliations:** 1 Laboratory of Clinical Examination and Sports Medicine, Department of Clinical Medicine, Faculty of Medicine, University of Tsukuba, 1-1-1 Tennodai, Tsukuba 305-8577, Japan; take0716@krf.biglobe.ne.jp (T.S.); yasukk1220@gmail.com (Y.K.); 2 Department of Sports Medicine Analysis, Open Facility Network Office, Organization for Open Facility Initiatives, University of Tsukuba, 1-1-1 Tennodai, Tsukuba 305-8577, Japan; srs8414@u.tsukuba.ac.jp; 3 Doctoral Program in Sports Medicine, Graduate School of Comprehensive Human Sciences, University of Tsukuba, 1-1-1 Tennodai, Tsukuba 305-8577, Japan; 4 Faculty of Health and Sport Sciences, University of Tsukuba, 1-1-1 Tennodai, Tsukuba 305-8574, Japan; watanabe.koichi.ga@u.tsukuba.ac.jp

**Keywords:** doping, blood, autologous blood transfusion, RNA sequencing

## Abstract

The World Anti-Doping Agency (WADA) has prohibited the use of autologous blood transfusion (ABT) as a doping method by athletes. It is difficult to detect this doping method in laboratory tests, and a robust testing method has not yet been established. We conducted an animal experiment and used total RNA sequencing (RNA-Seq) to identify novel RNA markers to detect ABT doping within red blood cells (RBCs) as a pilot study before human trials. This study used whole blood samples from Wistar rats. The whole blood samples were mixed with a citrate–phosphate–dextrose solution with adenine (CPDA) and then stored in a refrigerator at 4 °C for 0 (control), 10, or 20 days. After each storage period, total RNA-Seq and bioinformatics were performed following RNA extraction and the purification of the RBCs. In the results, clear patterns of expression fluctuations were observed depending on the storage period, and it was found that there were large numbers of genes whose expression decreased in the 10- and 20-day periods compared to the control. Moreover, additional bioinformatic analysis identified three significant genes whose expression levels were drastically decreased according to the storage period. These results provide novel insights that may allow future studies to develop a testing method for ABT doping.

## 1. Introduction

Doping is the act of using prohibited substances and/or methods in sports to enhance athletic performance [1]. The World Anti-Doping Agency (WADA), which was established in 1999, is involved in scientific research on doping, anti-doping education, the development of anti-doping strategies, and the monitoring of the World Anti-Doping Code to ensure soundness and fairness in sports worldwide [2]. However, despite the WADA’s substantial efforts, doping has not been eradicated from competitive sports. The International Standard Prohibited List [3], which is stipulated in the World Anti-Doping Code 2022 [4] and published with annual revisions by the WADA, describes various formulations and methods used in doping. This list includes “autologous blood transfusion (ABT)” in “M1. MANIPULATION OF BLOOD AND BLOOD COMPONENTS.” ABT is a collection of blood from a single patient and retransfusion back to the same patient when a large amount of bleeding is expected due to surgery that is highly invasive, such as orthopedic, cardiovascular, or cerebrovascular surgery [5,6,7,8]. This is in contrast to allogenic blood transfusion, where blood from unrelated/anonymous donors is transfused to the recipient, which has many advantages for patients in need of transfusions. The advantages of ABT compared to homologous blood transfusion (HBT) are that it reduces the risk of infection and immune response, and it can be provided at a lower cost because it eliminates the expensive preparation process [5]. Therefore, it has been recognized as the safest blood transfusion method. Moreover, because the blood of the individual is returned to the same individual without artificial compounds and hormone agents, it is considered very difficult to detect proof of it being used as doping in athletes. Furthermore, there is scientific evidence that ABT increases exercise performance over a broad range of exercise intensities and durations [9,10]. For these reasons, there are still serious concerns that ABT is abused as a doping strategy by athletes. Real examples include the abuse of ABT by athletes who participate in famous cycling competitions, such as the Tour de France [11,12]. In addition, most recently, a Nordic skier who participated in the Nordic World Ski Championships was exposed and then arrested on suspicion of ABT doping by police officers in Austria [13]. It is predicted that there are many cases of ABT doping that evade blood tests. It seems that a robust method that can directly detect ABT doping has not yet been established internationally. For that reason, the WADA is currently offering a research grant for research and development toward methods for detecting ABT doping [14]. In previous studies, attempts have been made to develop a method for detecting proof of ABT using blood or urine [15,16,17,18,19,20]. However, since the marker used by that method is still in the range of detection for indirect proof, it may be preferable to develop robust and direct detection methods in the future.

We theorized that a method similar to the test for doping via HBT [21,22] would be the most rational way to detect the molecular signature of ABT in a single red blood cell (RBC). In particular, the analysis of RNA molecules in RBCs had not been reported in research in this field, and we thought it could provide new insights. Therefore, in this study, we focused on RNA molecules in rat RBCs as a pilot study. The RNAs were comprehensively analyzed in terms of the expression fluctuations that depended on the blood storage period. Since we identified candidate RNA markers in RBCs, this study may provide novel insights that allow future studies to identify ABT markers in humans.

## 2. Materials and Methods

### 2.1. Animal Experiments

Animal experiments in this study were approved by the Animal Care Committee, University of Tsukuba (approval number: 21-016). Six male 10-week-old Wistar rats were purchased from Japan SLC (Hamamatsu, Shizuoka, Japan) and then subjected to a 1-week acclimation period. The rats were bred and maintained in an air-conditioned animal house under specific-pathogen-free (SPF) conditions and subjected to a 12/12 h light and dark cycle. The rats were fed standard mouse pellets and water ad libitum during the acclimation period. At the start of the experiments, the rats were 11 weeks old, and their body weights were 261–280 g. At 11 weeks, 10 mL syringes containing 1 mL of citrate–phosphate–dextrose solution with adenine (CPDA) (Karmi CA solution; SB-KAWASUMI, Kawasaki, Kanagawa, Japan) were prepared. Next, approximately 7 mL of whole blood from the inferior vena cava of the rats was harvested under systemic inhalation anesthesia with isoflurane, followed by euthanasia by bleeding from the abdominal aorta. The harvested whole blood was dispensed into a 15 mL centrifuge tube and was mixed well, which finally became 8 mL including 12.5% CPDA. The blood samples were quickly placed on ice; then, each was aliquoted into three equal volumes in three new 15 mL tubes.

### 2.2. Storage Conditions for the Whole Blood

The whole blood samples (including CPDA) were stored for 10 or 20 days (10 d or 20 d) at 4 °C in a refrigerator. After the passage of time in each condition, the blood was subjected to the next purification step. The control condition (Cont) was also considered as non-storage and was subjected to the next purification step on the day of blood sampling. The number of samples was N = 6 as paired samples under each storage condition.

### 2.3. Purification of Red Blood Cells

**Figure 1:**
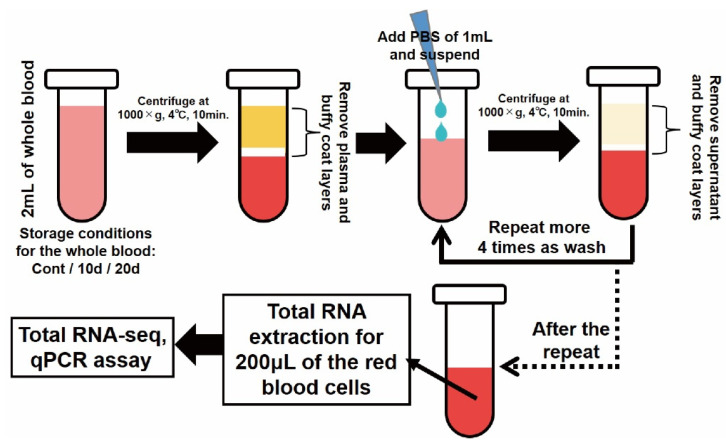
Purification method for RBCs in this study.

Since this study targeted RBCs, it was necessary to purify them. The RBCs were purified by washing the whole blood several times as follows. Briefly, 2 mL of the stored blood was suspended in a 2 mL micro tube, then centrifuged at 1000× *g* and 4 °C. Next, plasma and buffy coat layers were removed. Then, the blood was resuspended using 1 mL of PBS. Centrifugation was performed again under the same conditions to remove the supernatant containing the diluted plasma and buffy coat before the washing step. This washing step was repeated 4 more times; in total, washing was performed 5 times. An overview of the purification method for RBCs is shown in Figure 1. After purification, the RBCs were subjected to a further preparation step. As a supplementary experiment, the degree of purification for RBCs obtained by this method was measured using quantitative real-time PCR (qPCR) assay on the normal whole blood (including the CPDA) of the 5 rats. See Section 2.5.

### 2.4. RNA Extraction and cDNA Synthesis

RNA extraction for all samples was performed using 200 μL of the purified RBC with RNAiso Blood (Cat#9112, Takara Bio, Kusatsu, Shiga, Japan), according to the manufacturer’s instructions. The extracted total RNA solution in Milli-Q water (30 μL) was diluted and adjusted to a concentration of 100 ng/uL. The RNAs were pooled within each time point, and the degree of degradation was confirmed using an Agilent RNA 6000 Nano Kit (Cat# 5067-1511; Agilent Technologies, Santa Clara, CA, USA) on a Bioanalyzer (Agilent Technologies). Next, 500 ng of RNA was used to make cDNAs with the PrimeScript RT Master Mix (Cat#RR036A, Takara Bio), according to the manufacturer’s instructions. The cDNAs, as templates, were diluted at ×10 using Milli-Q water and subjected to qPCR assay based on SYBR Green dye. The extracted total RNA was also subjected to total RNA sequencing (RNA-Seq).

### 2.5. qPCR Assay

To confirm the purification level of the RBCs, the gene expression of *Ptprc* (CD45) was measured using qPCR as preliminary verification (Supplementary Figure S1). In addition, expression of the candidate RNA markers found by the total RNA-Seq, which is described later, was also measured by qPCR to confirm accuracy for the RNA-Seq results. All samples (N = 6 for each condition) were subjected to qPCR assay. The primer sequences used in this assay are shown in Supplementary Table S1. The preparation and quantification methods were as follows. First, the reaction plate was prepared as follows: 2 μL of template cDNA, 0.1 µL of a 10 µM primer solution (forward and reverse each), 5 µL of master mix (KAPA SYBR FAST qPCR kits, Cat# KK4620; NIPPON Genetics, Bunkyo, Tokyo, Japan), and 2.8 µL of Milli-Q water were included for a total reaction volume of 10 μL per well on a qPCR plate. Negative control wells were also prepared using Milli-Q water in the assays instead of a template. Then, amplified reactions were performed on a QuantStudio 5 Real-Time PCR System (Thermo Fisher Scientific, Waltham, MA, USA) in thermal cycle conditions as follows: one cycle of 95 °C for 5 min, followed by 40 cycles of 95 °C for 3 s and 60 °C for 30 s, and a final stage of melting curve analysis. All qPCR assays were conducted as duplicate measurements. Subsequently, the ΔΔCt method referencing *Rpl13a* was used to calculate the relative gene expression values.

### 2.6. Total RNA-Seq

Total RNA-Seq was performed to identify the novel RNA markers, that is, fluctuated expressions dependent on the blood storage period. The method was similar to procedures in our previous study [23,24]. Preparation of the libraries and NGS run was conducted at the Department of Sports Medicine in the Organization for Open Facility Initiatives, University of Tsukuba (Tsukuba, Ibaraki, Japan). A total of 12 RNA samples, N = 4 for each condition (i.e., Cont, 10 d, and 20 d), were examined in terms of degradation using an Agilent RNA 6000 Nano Kit (Cat# 5067-1511; Agilent Technologies) on a Bioanalyzer (Agilent Technologies). After examination and after passing quality checks, all RNA samples were subjected to library preparations for the RNA-Seq. Using 500 ng of the total RNA from each sample, libraries were created using the NEBNext Ultra II RNA Library Prep Kit for Illumina and the NEBNext rRNA Depletion Kit (Cat# E7770 and E6310; New England Biolabs, Ipswich, MA, USA), according to the manufacturer’s instructions; the final PCR cycle was number 12. Concentration and size distributions of the libraries were measured using an Agilent DNA 7500 kit (Cat# 5067-1506; Agilent Technologies) with a Bioanalyzer. All samples were subjected to analyses on NGS equipment. The libraries were pooled, and the concentrations adjusted to 1 nM. The pooled libraries were subjected to denaturation and neutralization. Subsequently, the libraries were diluted to 1.8 pM and then used for an NGS run using NextSeq 500/550 v2.5 (75 cycles) kits (Cat#20024906; Illumina, San Diego, CA, USA) in a NextSeq 500 System (Illumina). The sequencing was performed with paired-end reads of 36 bases. After the sequencing run, FASTQ files were exported, and the basic information of the NGS run data was checked by CLC Genomics Workbench 22.0 software (QIAGEN, Venlo, Limburg, The Netherlands). In the quality assessment of the reads, a PHRED score over 20 was confirmed for 99.53% of all reads, indicating the success of the run. The read number was approximately 71.9 million to 92.2 million per sample as paired-end reads.

### 2.7. Bioinfomatic Analysis

Bioinformatic analysis was performed to understand the expression profile and to identify novel RNA markers. The FASTQ files were mapped to the rat reference genome (mRatBN7.2) with an annotation file (mRatBN7.2.105) using the tool, “RNA-Seq analysis,” in CLC software. Then, expression values of all genes were obtained as CPM (counts per million) and TPM (transcripts per kilobase million). Statistical differential expression analysis was conducted using the tool, “Differential Expression for RNA-Seq,” as an ANOVA-like test based on the likelihood ratio test in CLC software. The expression values and results of the statistical tests are shown in Supplementary Table S2. Differentially expressed genes (DEGs) were considered with a false discovery rate (FDR) < 0.0001 on the ANOVA-like test in CLC software. The heat map, including clustering analysis (the setting was Distance measured = Euclidean distance; Linkage criteria = Average linkage), was created using the DEGs in CLC software. A principal component analysis (PCA) plot was also created with CLC software using expression values for all genes. A cluster dendrogram with Ward’s minimum variance method was also created through R software (version 4.1.1) using expression values for all genes. Box plot and plot graphs were created using GraphPad Prism software (version 9.3.1; GraphPad, San Diego, CA, USA). Z-scores calculated from Log_10_ (CPM+1) were used to create the box plot and perform statistical analysis. The scatter plot was created using averaged TPM values + 1 on Excel software (Office 2019; Microsoft, Redmond, WA, USA). A bar graph was also created with Excel software. A Venn diagram was created with the web tool, “Venny version 2.1” [25].

### 2.8. Statistical Analysis

Statistical analysis for data, excluding data tested by CLC software, was performed using GraphPad Prism software (version 9.3.1). The normality of the distributions was evaluated with the Shapiro–Wilk normality test. Subsequently, nonparametric tests were used for the box plot data (non-normal distribution), to which was applied Kruskal–Wallis H tests (one-way ANOVA of ranks), followed by a two-stage Benjamini, Krieger, and Yekutieli FDR procedure as a post hoc test. Parametric tests were used for the plot graph data (normal distribution), to which was applied one-way ANOVA (repeated measures) with Greenhouse–Geisser correction followed by a Tukey’s multiple-comparison post hoc test. A *p*-value less than 0.05 was considered to indicate statistical significance.

## 3. Results

### 3.1. There Are Some Changes in the Electrophoretic Patterns of RNA from RBCs

**Figure 2:**
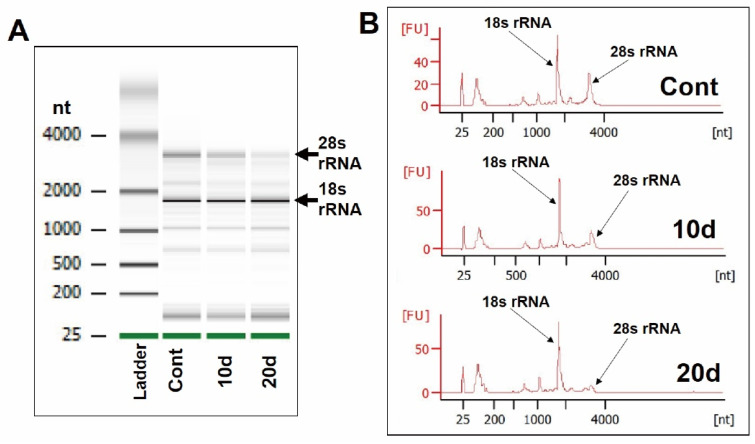
Electrophoresis patterns of the RNAs from RBCs in each storage condition. (**A**) Gel image and (**B**) histogram in the Bioanalyzer.

Even though the electrophoresis gel images of RNAs extracted after each storage condition were approximately same, the 28 s ribosomal RNA (rRNA) band for the 20 d sample was attenuated compared to the Cont and 10 d samples (Figure 2A). Similarly, the peak signal of the 28 s rRNA was lower than for other conditions in the histogram (Figure 2B). From these results, it can be considered that no storage condition caused severe degradation of RNAs, which would have led to smeared bands. There were also sufficient amounts of RNAs extracted from the RBCs (average μg ± SD: 8.03 ± 1.44). Therefore, it was determined that these RNAs could be subjected to the total RNA-Seq.

### 3.2. Total RNA-Seq Revealed Differential Profiles for Each Storage Condition

**Figure 3:**
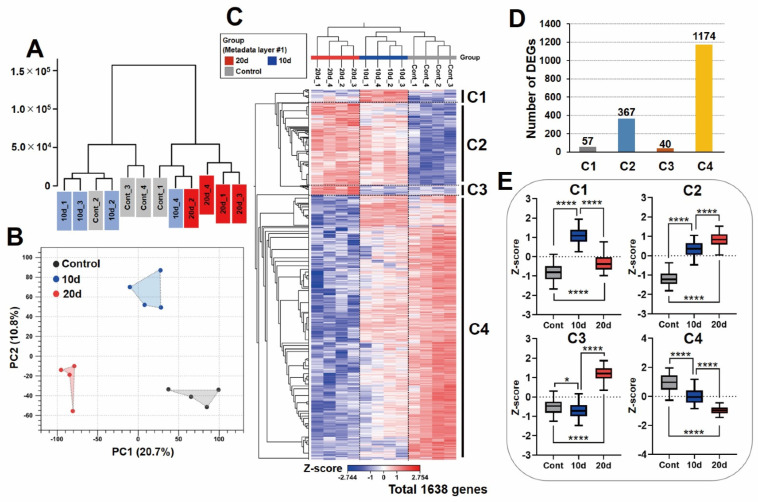
Overall expression profiles of the three storage conditions. (**A**) Phylogenetic tree, (**B**) PCA plot, (**C**) heat map filtered by FDR *p*-value < 0.0001 in ANOVA-like test, (**D**) number of DEGs in the clusters, and (**E**) box plot for each cluster. C1–C4: clusters 1–4. * *p* < 0.05, **** *p* < 0.0001.

Figure 3 shows the overall expression profiles of the three storage conditions. The phylogenetic tree suggested that there were differential expression patterns among the three storage conditions (Figure 3A). In particular, 20 d seemed to distinguish itself from Cont and 10 d. The PCA plot shows clear separation for each storage condition, supporting results in the phylogenetic tree (Figure 3B). From the results so far, it can be inferred that there are significant differences in RNA expression among the three storage conditions. Figure 3C shows the heat map, which was filtered by FDR *p*-value < 0.0001 with an ANOVA-like test. There were significant differences in 1638 genes among the storage conditions, as shown on the heat map (Figure 3C). Moreover, the phylogenetic tree (*y*-axis) for each sample clearly separates each storage condition on the heat map, which is supported by the results of the phylogenetic tree (Figure 3A) and PCA plot (Figure 3B). Additionally, the phylogenetic tree for the gene expression patterns (*x*-axis) could be divided into four clusters (C1–C4; Figure 3C) on the heat map. The DEGs numbers in C1–C4 are shown in Figure 3D, and C4 contained the greatest number of genes that fluctuated in expression. Finally, box plots in Figure 3E show the fluctuating patterns of gene expression in each cluster. Similar to the color changes in the heat map (Figure 3A), a statistically significant fluctuation pattern was confirmed (Figure 3E; C1–C4). In particular, since C2 and C4 showed unidirectional fluctuations in expression, it was considered that there are novel RNA markers that can reflect storage conditions in these clusters. Taken together, as the results of these bioinformatic analyses are mutually consistent, we considered it reasonable to carry out further analysis because the accuracy can be guaranteed. Therefore, additional bioinformatic analysis was performed in detail.

### 3.3. Identification of Novel RNA Makers in RBCs

**Figure 4:**
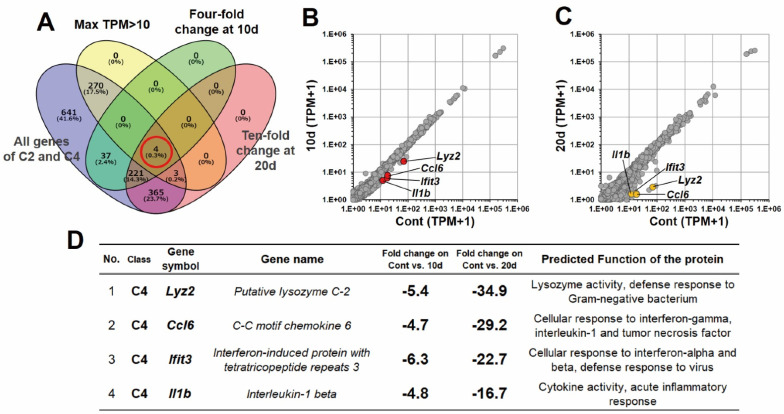
Identification of four candidate RNA markers in RBCs. (**A**) Venn diagram including conditions as follows: 4-fold change in averaged TPM values at 10 d, 10-fold change in averaged TPM values at 20 d, and maximum averaged TPM value > 10. (**B**) A scatter plot between Cont and 10 d. (**C**) A scatter plot between Cont and 20 d. The color dots show 4 genes of the candidate RNA markers obtained from the Venn diagram analysis. (**D**) A table including each characteristic of each candidate RNA marker.

Additional bioinformatic analysis was performed focusing on C2 and C4. Venn diagram analysis was undertaken to identify novel RNA markers using gene lists of C2 and C4, with the following conditions: 4-fold change in averaged TPM values at 10 d, 10-fold change in averaged TPM values at 20 d, and maximum averaged TPM value > 10. In the results, four RNAs were matched in the Venn diagram (Figure 4A; central red circle), which we believe to be novel RNA markers in a rat model. When the expression levels of these four RNAs were visualized in the scatter plots, the levels for both 10 d and 20 d were lower than for Cont (Figure 4B,C). The rate of expression reduction increased between 10 and 20 d, compared to 0 to 10 d. Figure 4D shows cluster classification, gene symbols, fluctuation rates, and functions of the novel RNA markers in a table. Since the table data are consistent with the other figures, these bioinformatic analyses were performed correctly. Taken together, these results suggest that the four genes are efficient as novel RNA markers for ABT in rat models.

### 3.4. qPCR Assay Showed Reproductivity of Expression Fluctuation Patterns for Lyz2, Ifit3, and Il1b

**Figure 5:**
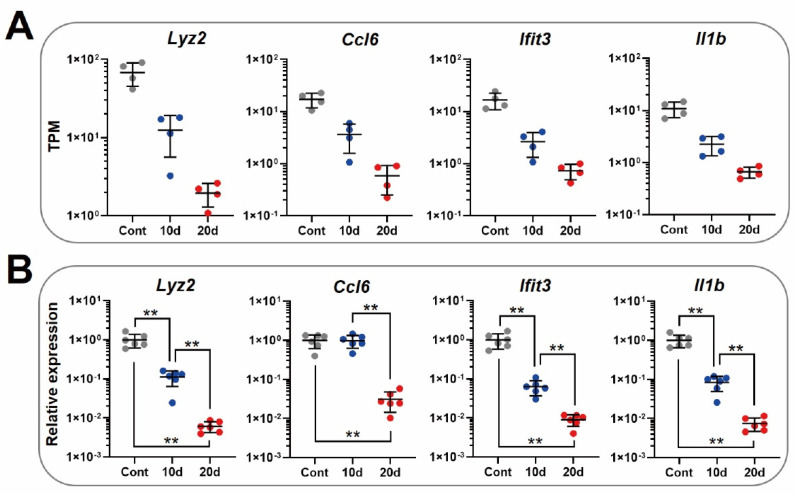
Confirmations of reproducibility using the qPCR assay. (**A**) Plot graphs of the TPM values from the RNA-Seq. (**B**) Plot graphs of the relative expression values from the qPCR assay. ** *p* < 0.01.

qPCR assays were performed using all samples to confirm the reproducibility of the RNA-Seq data. After the qPCR assay, the TPM values obtained from the RNA-Seq and the relative expression values obtained from the qPCR assay were visualized as plot graphs (Figure 5). The results confirmed that the relative expression values of *Lyz2*, *Ifit3*, and *Il1b* had similarly fluctuated patterns to the TPM values: the expression level decreased according to the storage period. On the other hand, the relative expression patten and TPM were not similar for *Ccl6*. Taken together, these results suggest that *Lyz2*, *Ifit3*, and *Il1b* are novel and candidate RNA markers for detecting ABT doping in a rat model.

## 4. Discussion

Blood doping is considered when athletes abuse recombinant erythropoietin (EPO), erythropoiesis stimulating agents, or blood transfusion to gain a temporary enhancement in performance. Blood doping has frequently been used in the history of sports [26,27]. Focusing on the Olympics, many cases have been reported, from the 1972 Munich Olympics to the modern Olympics [27]. Recent cases include blood doping via HBT in relation to a track and field athlete at the Tokyo Olympics of 2020 [28], and ABT use by a Nordic skier at the Nordic skiing world championships in 2019 [13]. Considering these cases and sports history, it seems that, even with the development of modern science and technology, there have probably been many undetectable cases of blood doping. In particular, ABT has a history of being abused by athletes [11,12,13,26]. However, cases have been discovered when a player confessed or a police officer found evidence, but a robust testing method for ABT doping has not yet been established. Its detection is difficult [26] because the testing method must detect changes in blood components from the same individual, not artificial components. Therefore, the establishment of a testing method has been an urgent task to eradicate potential ABT doping. Thus, based on a new concept, this study conducted experiments aimed at establishing a laboratory test method for ABT doping in the future.

A method for detecting HBT doping has already been established, which involves testing the antigens of individual red blood cells with a flow cytometer [21,22]. In this research, based on the concept of the test for HBT doping, we considered marker candidates that can be directly detected. Since it seemed reasonable to focus on RNA in RBCs because there were no reports on this method in previous studies, rat blood was stored in CPDA for 10 days or 20 days, and RNA in RBCs was comprehensively analyzed to identify novel marker RNAs. In the results for the RNA extraction of the RBCs, the RNA was abundant and confirmed by 18s and 28s rRNA bands (Figure 2), unique to mammalian tissue. It was also confirmed that there was no band with smearing [29], and intact states without 28s rRNA bands were confirmed in each storage condition (Figure 2). Therefore, we concluded that the RNA in the RBCs was stable and unaffected by the storage conditions and consistent with previous reports of human erythrocytes [30,31]. RBCs undergo denucleation during their maturation from blood stem cells before entering the peripheral blood [32,33]. It can also be speculated that the RNA in the RBCs is generated before denucleation. However, there seems to be no previous study that provides clear answers. This question may need to be investigated during the future development of a test for ABT doping.

Next, total RNA-Seq was performed using the RNA to obtain an expression profile for each storage condition. It was revealed that the expression values of many genes decreased when extending the storage period (Figure 3C, C4; 1174 genes). This was particularly noticeable for the 20-day storage period. These results suggest that certain RNAs degrade depending on the storage period. On the other hand, genes whose expression increases depending on the extended storage period were also confirmed (Figure 3C, C1, 2, and 3; 464 genes). Since there is no nucleus and no RNA expression in RBCs, this is an incomprehensible phenomenon at first glance.

Considering the RNA and protein signaling tools between cells, the exosome secretion mechanism should be the answer to this otherwise incomprehensible phenomenon. Recently, exosomes have become a rapidly developing topic in molecular biology. Exosomes, as extracellular vehicles, are secreted through exocytosis by all living cells (donor cells) to recipient cells and contain diverse bioactive molecules: RNA (mRNA, miRNA, and non-cording RNA), proteins, and lipids [34,35,36]. Then, the cells, receiving the exosomes via endocytosis, undergo various biological changes depending on their contents [34,35,36]. In this study, it was inferred that the purified RBCs did not have any nuclei, and there was almost no expression of new RNAs. On the other hand, WBCs in whole blood during the storage period had nuclei, and the gene expression undeniably fluctuated in CPDA. Taken together, it is possible that, during the storage period for whole blood, gene expression in WBCs was altered, and RNAs were transferred to the RBCs via exosomes. Furthermore, the storage period or environment could affect specific biological processes in WBCs and RBCs. Therefore, further investigation of these phenomena may provide insights for the development of a new test for ABT doping. However, in the Venn diagram (Figure 4A), 464 genes did not have high expression or fluctuations, so we did not focus on them in this study. The next task in our studies will be to thoroughly investigate those 464 genes and exosomes.

The novel candidate marker RNAs were the *Lyz2*, *Ifit3*, and *Il1b* genes finally identified in this study (Figure 5). The database references show that these genes are associated with the immune system; however, their significance in RBCs remains unclear. The expression levels of these RNAs decreased during storage. In particular, the expression levels were drastically deceased after 20 days of storage compared to the control or 10 days of storage. Moreover, the maximum TPM value was confirmed to be over 10 for each RNA. If researchers want to establish a method for ABT doping that targets RNAs in RBCs, a marker with high expression (TPM > 10) and high fluctuation (over 10-fold change) may be suitable. This is because the probability of false negatives and false positives may be significantly reduced in the calculation of sensitivity and specificity with the receiver operating characteristic (ROC) curve that will be established in the future. Therefore, if the three candidate RNAs are targeted after the creation of the ABT doping model for rats, a testing method to detect ABT doping in humans may be developed.

At present, a testing method for HBT doping is based on the technology of flow cytometry [21], and it has been used in various international competitions. In addition, more accurate methods have recently been developed [22]. Since flow cytometry can detect signals from individual RBCs and can accurately detect doping, it is hoped that the results of this study can be exploited by flow cytometry as well. However, there are some technical problems in applying flow cytometry. It is generally very difficult to measure the molecules inside red blood cells. When RBCs are processed in terms of permeabilization and fixation, which is generally done, they instantly become hemolytic and cannot maintain their morphology. This probably depends on the peculiarities of the membrane structure of RBCs. Healthy RBCs deform readily in response to mechanical forces in blood flow, facilitating their efficient passage through capillaries [37,38]. RBCs, compared with other cells in the human body, are particularly simple in structure [38]. The membrane of an RBC consists of two components: a lipid bilayer and an attached spectrin-based skeleton (cytoskeleton). These two components are connected by transmembrane proteins, such as band-3 and glycophorin C [38]. Furthermore, the lipid bilayer is considered to be nearly viscous and area-preserving, and the spectrin-based network is largely responsible for the elastic properties of RBCs [38,39]. Therefore, it is possible that these characteristics are factors in the fragility of RBCs in the preparation for flow cytometry. In order to adapt RBC analysis to flow cytometry, it will be necessary to develop a new preparation method that takes this characteristic into consideration.

There were some limitations to this study. Since it was an experiment using rats, the results cannot be directly applied to human studies. In addition, this study only conducted comprehensive analysis of the RNAs in RBCs and did not establish a testing method for ABT doping within a rat model. It would be strongly desirable to develop a method that can quantify RNAs in individual RBCs by flow cytometry. Furthermore, because there would be an exosome-mediated effect during the storage period in the transfusion bag, molecular mechanisms may need to be revealed in detail. After that, creating an ABT model in rats would be needed to examine the mechanisms in detail and develop a testing method. Nevertheless, this study identified candidate marker RNAs that significantly fluctuate according to the storage period. Therefore, the findings in this study could provide a major step toward future research.

## 5. Conclusions

In this study, the RNA in the RBCs after each storage condition was comprehensively analyzed to identify novel RNA markers to detect ABT doping using total RNA-Seq. As a result, we identified three RNA markers with drastically decreased expression levels after storage. Therefore, it is expected that the identified RNA markers and the methods used in this study will be useful for further studies to develop testing methods for ABT doping.

## Data Availability

The total RNA-Seq data as FASTQ files and an expression browser as table data have been deposited in the “Gene Expression Omnibus (https://www.ncbi.nlm.nih.gov/geo/; accessed on 13 June 2022)” under accession number: GSE205806.

## Supplementary Materials

The following supporting information can be downloaded at: https://www.mdpi.com/article/10.3390/genes13071255/s1. Figure S1: Confirmation of purified degrees for RBCs by the qPCR assay. Table S1: primers. Table S2: expression values and statistics in the RNA-Seq.
