# Supplementary material for: Identification of RNA Markers in Red Blood Cells for Doping Control in Autologous Blood Transfusion
Source: Genes (Basel). 2022 Jul 15;13(7):1255. doi: 10.3390/genes13071255 (PMC9317427; doi:10.3390/genes13071255)
Supplement: Supplementary file 1 [file genes-13-01255-s001.zip › Figure S1.pdf]

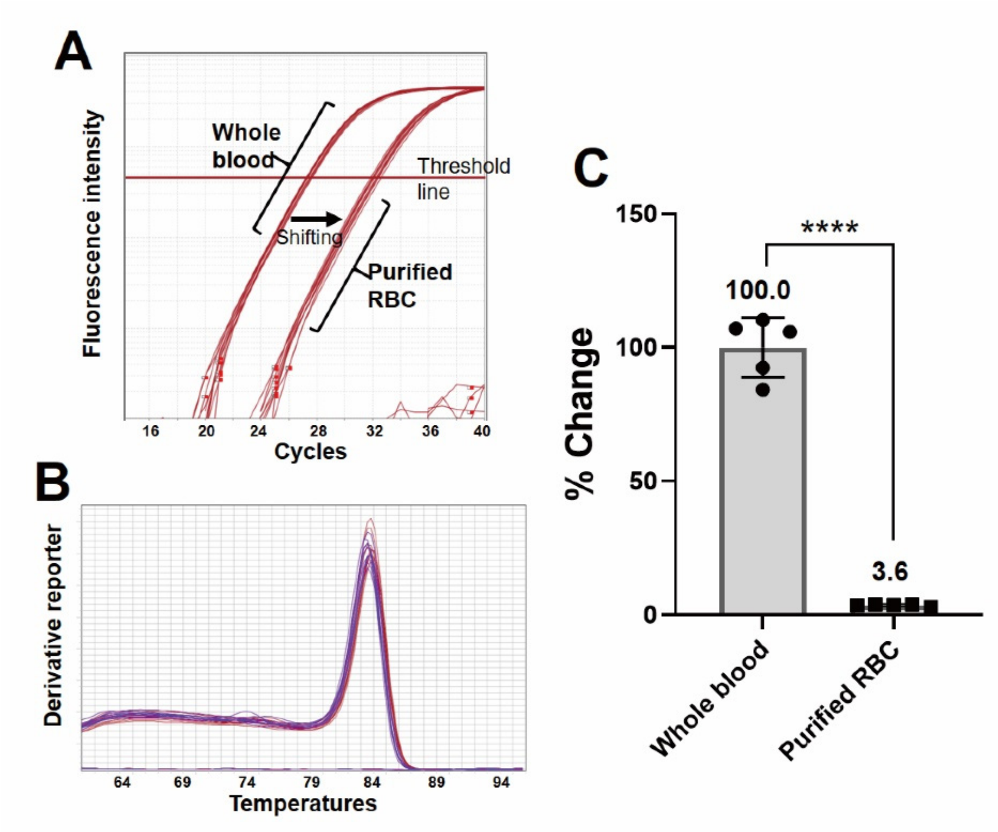

Supplementary Figure S1. Confirmation of degree of purification of RBCs by the qPCR assay. A primer pair for *Ptprc* (CD45) was used in this assay. (**A**) Amplification plot of all samples. The amplification curves of whole blood samples that shift to the right side from the left side accompanied purification of RBC. (**B**) Melt curve plot shows one peak signal, which means the primer pair was specific to the target gene. (**C**) Bar graph showing %change for the relative expression value of the *Ptprc* gene. After purification of RBCs, the expression was decreased to about 1/28 on average, which means that the purification was performed sufficiently well. **** $p < 0.0001$ on paired t-test.
